# Planning and establishment of a high throughput screening site

**DOI:** 10.1155/S1463924698000108

**Published:** 1998

**Authors:** Julie J. Tomlinson, Brent T. Butler, Joan Frezza, Cole O. Harris, Albert A. Smith, W. Blaine Knight

**Affiliations:** Molecular Biochemistry Glaxo Wellcome Research Triangle Park NC 27709 USA

## Abstract

In 1996 and 1997, Glaxo Wellcome's US Research division planned and established their second generation research strategy. An important aspect of the strategy entailed development of two automated screening sites in Biochemistry in Research Triangle Park, NC. Development of the new operations required many decisions to be made very quickly, including automated process design, system selection and site preparation. Descriptions of the decision made in the development of one of the screening sites are presented in this paper.


Journal of Automatic Chemistry, Vol. 20, No. 3 (May–June 1998) pp. 83–85

### Planning and establishment of a high throughput screening site

**Julie J. Tomlinson, Brent T. Butler, Joan Frezza, Cole O. Harris, Albert A. Smith and W. Blaine Knight**

*Molecular Biochemistry, Glaxo Wellcome, Research Triangle Park, NC 27709, USA*

*In 1996 and 1997, Glaxo Wellcome's US Research division planned and established their second generation research strategy. An important aspect of the strategy entailed development of two automated screening sites in Biochemistry in Research Triangle Park, NC. Development of the new operations required many decisions to be made very quickly, including automated process design, system selection and site preparation. Descriptions of the decision made in the development of one of the screening sites are presented in this paper.*

### Introduction

During 1996 and 1997, scientists at Glaxo Wellcome's (GW) Research Triangle Park, North Carolina (RTP) site planned and implemented their second generation US Research Strategy. This strategy called for the development of more efficient processes to help ensure that the world-wide corporation achieves its overall goals for the coming years. Thirteen workgroups of scientists were formed in early 1996 to plan the new processes. Each workgroup focused on a specific area, such as automation, data handling, lead optimization or compound distribution. The workgroups were small, usually six to 10 scientists selected from across research, development and information technology. In all, over 100 scientists participated in formulating the plans.

The workgroups were given three months to compile recommendations and submit reports to senior management. They formulated recommendations through collection and analysis of existing data, intra- and inter-group discussions, project meetings, and interviews with and feedback questionnaires from scientists through the organization.

When they received the 13 reports, senior management studied them and devised one overall strategy. Within one month the managers and scientists started the implementation of the second generation strategy. Within another month, implementation of the strategy was moving at full speed. Since then the original recommendations from the 13 workgroups have provided the backbone for on-going implementation.

A very large part of the strategy focuses on the processes surrounding high throughput primary screening (HTS) and secondary screening, including the processes that feed into them (for example compound distribution, assay development), the processes that they feed into (for example data handling, hit identification), and the automation of many of those processes.

Many of the final workgroup reports recommended that two centralized automated screening sites be developed in RTP. This recommendation became an integral part of the ensuing implementation effort. Both screening sites are in departments in US Biochemistry and reside in separate buildings: one site is in a main R&D complex and the other is in a satellite building several miles away from the main site. The development of the site in the satellite building is described herein.

Many decisions were made during site development, including workflow (processes and organization) and resources (space, people and instruments). All decisions were made and implemented in less than a year. Some decisions were easy to make; others were difficult. The more difficult or complicated decisions are the focus below.

### *Site functions*

Most of the functions of automated screening site were predetermined by, and fall within, the scope of the department: primary target screening to identify hits and secondary screening to confirm and evaluate hits. The decision to add compound handling functions was made three months into the planning process. Compounds received from a central inventory and distribution unit are prepared for the assays at the screening site.

To decide how we would automate these processes required decisions on:

- site processes and organization;
- selection and purchase of instruments;
- construction of space for automated systems.

### Site processes and organization

### *Processes and organization—options*

Automated screening processes are performed and groups are organized in many different ways across Glaxo Wellcome and the pharmaceutical industry. Process flow is largely determined by the way that people are organized, even though the reverse scenario is usually the preferred one. While screening processes were being developed at this site, the following options for organization were available: organization of automation expertise could be centralized or non-centralized and organization of assay operations could be centralized or non-centralized.

*Centralized automation expertise:* centralized groups of scientists that are dedicated to setting up automated systems are very common in laboratory automation. The greatest benefit to this arrangement is that it recognizes the significant level

0142-0453/98 $12·00 © 1998 Taylor & Francis Ltd of focused effort required to set up and maintain most automated systems. Its largest pitfall is the workflow: full centralization places boundaries in workflow, particularly between the system developers and the assay developers.

*Centralized screen operation*: centralized groups that are dedicated to running screens are also common; often it is the same group that has centralized automation expertise. The benefit of this arrangement is speed and efficiency: the screens operate as assembly-line processes. The downside to this arrangement is that, while it eliminates boundaries within screen conduct, it places stricter peripheral boundaries. For instance, the original assay developers, the primary screeners and the secondary screeners are often in different departments or divisions, and assays are developed as each of those boundaries is crossed. The ease by which those boundaries are crossed depends on the quality of communication and commonality of goals, which often depend on political organization. The closer these groups are situated politically in the organization, the easier it is to work across the boundaries.

*Non-centralized automation expertise*: non-centralized automation expertise often develops where a decision to automate is made locally and is not part of a strategic initiative in which resources are made available for personnel, space, etc. The benefits of this arrangement are that it is less expensive (in the short term), and it can be a very good way for an organization to get started with and learn about automation. The pitfall is that successful implementation often takes too long to achieve and confidence in benefits of automation is eroded. If it is successful, expansion is more difficult, because space, personnel and workflow to support expansion are not adequately pre-planned.

*Non-centralized screen operation*: non-centralized system operation is also sometimes referred to as walk-up operation. People use the system when they have an assay to run, rather than handing the assay off to a central screening group. A great benefit to this scheme is that the assay hand-off is eliminated. The major risk in this scheme is that the operators often know little about the systems and rely on others to set up and trouble-shoot them. It works best with readily available, local expertise.

*Process and organization—decisions*

It was decided that a core group of laboratory automation experts should be formed in the screening department to be physically and politically teamed with the scientists who develop the target screens. To avoid hierarchical segregation and to foster buy-in to the new strategy quickly, it was next decided that all scientists in the department who have targets to screen would be involved in screening them. Thus a centralized automation group was formed to develop, set up, and maintain automation in the department and that would work with scientists (known as target co-ordinators) to automate the target assays. The target co-ordinators would be responsible for developing the assays, supplying reagents, loading and running the systems and handling data.

### Selection and purchase of instruments

*Automated instruments—options*

Amongst the scientists and laboratories of Glaxo Wellcome is extensive experience with most types of laboratory automation software and hardware. Information on the performance of the many different systems and vendors is available from GW colleagues all over the world. From this information it may be concluded that a skilled, focused automation group would be able to maintain and operate virtually any type of system available, or could learn to do so. The deadline for site setup was challenging, therefore the realistic options available included systems that could be set up, operated and maintained quickly. This limited the systems to those that the people in the automation group and/or department and/or adjunct departments already knew how to set up, program and maintain. These systems are listed in table 1.

All instrument purchasing decisions were made from the options listed above.

*Automated instruments—decisions*

Execution of very high throughput screens was immediately assigned to full robotic systems. This decision was made to allow full automation of the assays and free scientists to work on other responsibilities, such as data handling, while the assays are running. Additional semi-automated workstations were installed in most laboratories to help scientists develop assays that are most easily and successfully scaled up to full robot systems. Secondary screening is also automated and the semi-automated workstations are also used to do this work. Scientists in the department underwent (and continue to undergo) training to operate the standalone instruments.

A typical assay automation scheme:

Tecan Genesis® ⇒ Full track robot ⇒ Tecan Genesis®
Assay development–Primary screening–Secondary screening

Decisions on the specific vendors to purchase the instruments from were based on existing expertise, instrument flexibility and vendor flexibility. Additionally, we decided not to use third-party integrators.

*Table 1.*

| Hardware | Software |
|---|---|
| Beckman/Sagian: ORCA®, Biomek® | Beckman/Sagian: MSD®, SAMI®, Bioworks® |
| Packard: Multiprobe® | Packard: EasyPrep® |
| Tecan: Genesis®, ORCA® | Scitech: WinClara®, Clara® |
| Zymark: Zymate® series | Tecan: Logic®, Integrator, Toolbox |
| | Zymark: EasyLab®, PCS® |

The semi-automated workstation decision was made based on flexibility and local expertise. A unit that could handle very low volumes of liquids in 96 and 384 format, provide cold storage for reagents, be easily customized for new racks and had the flexibility to be integrated with any new full robot system, was needed. At the time the decision was made, only one vendor could fulfil all of those requirements and be supported using internal expertise. Units that were purchased and installed before these decisions were made also are routinely used and maintained.

While deciding on which type of full robot system to purchase, the selection was narrowed to two vendors based on existing systems, existing expertise and the elimination of third-party integrators. The final determining factors were vendor responsiveness and flexibility to meet our needs for on-going service, support, training and technology development. One vendor offered these benefits immediately. Systems purchased and installed before these decisions were made also are routinely operated and maintained.

### Space for automated systems

*Space—options*

Like the groups and the workflow, space may be central or distributed. Centralized labs are commonly used for full robot systems, mainly because they require so much space. The space allocated to automation for this screening site was large and ideally located in the middle of the department; no options were available, but none was needed.

Possibilities for laboratory design were virtually unlimited and the decisions regarding the design of the lab that was built were numerous. The outcome is described below.

*Space—decisions*

Fortuitously, we were given a very large, unfinished space in which to build the central robotics laboratory. The space is located in the middle of the department, allowing easy access to all scientists. With the help of a large number and wide variety of facilities engineers, laboratory construction experts, cell-based assay experts, automation experts and assorted laboratory planners, a detailed set of plans was drawn up and construction was completed in less than 10 months.

The robotics laboratory was designed to ensure easy installation and continuous operation of robot systems and to allow the flexibility to adapt to future changes in technology and/or workflow. Utilities that were installed to support the systems included air, vacuum, nitrogen, helium, carbon dioxide and clean water, auxiliary lines were installed for future needs. The systems had network and telephone connections, and power back-up by both generator and uninterruptible power supply. Cell culture rooms, autoclave, cold storage, and consumables storage were built with easy access to the robots and their users in mind. Most reagents are stored in the assay developers' laboratories, but accommodations were made to ensure convenience to the robot systems. Most benches in the laboratory have wheels; only those with sinks and utility connections are fixed.

The laboratory environmental conditions were also important. The laboratory temperature is held in a narrow range that is slightly colder than ambient for the building; the temperature is maintained in that range for 24 hours. Freezers are also maintained in a narrow temperature range and are alarmed. Attention was paid to the aesthetics of the laboratory; colour selections for cabinets and walls were taken very seriously. We believe that because the people working there spend more of their time there than almost anywhere else that they should spend that time in a pleasant environment.

During the 10 months that the permanent laboratory was designed and built, a laboratory adjacent to it was modified to allow immediate installation of robot systems and automation of high throughput screens. Laboratory benches and cabinets were removed and utilities were installed to support full robot track systems; the laboratory was converted to support robots quickly. Additionally, laboratories throughout the department were modified to accommodate installation of standalone automated workstations.

### Results

Development and implementation of these site-specific plans started in May 1996 and were completed in March 1997; all except the construction of the new laboratory were completed before the end of 1996. All high throughput screening, all compound preparation and most secondary screening activities in the department were automated in that timeframe.

Projects continue to expand, people continue to be trained, processes continue to be improved and technology will continue to evolve. Goals for the near future are focused on continuous improvement of the processes developed in the first year. Quality, not quantity, is the current focus and cost containment is always important. Many projects are in place that will ensure the long-term success of screening at this site and of GW drug discovery.

### Acknowledgements

Many people contributed to the success of this project. The authors extend their thanks to the members of Research IR and Development IR for their on-going support in system integration and development, to management for providing the resources that are essential to allow the project to be successful, and to the automation vendors for their support and flexibility that are critical to our on-going success.

